# Supplementary material for: Investigating Photo-Degradation as a Potential Pheromone Production Pathway in Spotted Lanternfly, Lycorma delicatula
Source: Insects. 2023 Jun 13;14(6):551. doi: 10.3390/insects14060551 (PMC10299250; doi:10.3390/insects14060551)
Supplement: Supplementary file 1 [file insects-14-00551-s001.zip › insects-2382430-supplementary.pdf]

Table S1. List of synthetic standards used for verification of tentatively identified compounds from headspace volatiles of spotted lanternfly, *Lycorma delicatula*

KI[1]	Compound[2]	Source	Purity
<800	Pentan-1-ol	Sigma–Aldrich[3]	99.7%
800	Octane	Sigma–Aldrich	99%
802	Hexanal	Sigma–Aldrich	98%
866	Hexan-1-ol	Sigma–Aldrich	99%
887	Heptan-2-one	Sigma–Aldrich	98%
901	Heptanal	Sigma–Aldrich	≥95%
971	Heptan-1-ol	Sigma–Aldrich	98%
980	Oct-1-en-3-ol	Sigma–Aldrich	98%
986	Octane-2,3-dione	THJ[4]	75%
991	Octan-2-one	Sigma–Aldrich	98%
1003	Octanal	Sigma–Aldrich	99%
1024	Hexanoic acid	Sigma–Aldrich	≥99%
1029	2-Ethylhexan-1-ol	Sigma–Aldrich	≥99%
1070	Octan-1-ol	Sigma–Aldrich	≥99%
1091	Nona-2-one	Sigma–Aldrich	≥99%
1092	Undec-1-ene	Sigma–Aldrich	97%
1100	Undecane	Sigma–Aldrich	99%
1102	Heptanoic acid	Sigma–Aldrich	97%
1103	(Z)-Non-6-enal	Sigma–Aldrich	≥95%
1105	Nonanal	Sigma–Aldrich	≥95%
1161	(E)-Non-2-enal	Fisher Scientific[5]	≥95%
1172	Nonan-1-ol	Sigma–Aldrich	98%
1190	Methyl 2-hydroxybenzoate	Sigma–Aldrich	≥98%
1193	Dodec-1-ene	Sigma–Aldrich	95%
1195	Octanoic acid	Sigma–Aldrich	98%
1207	Decanal	Sigma–Aldrich	≥98%
1264	(E)-dec-2-enal	Sigma–Aldrich	≥97%
1291	Nonanoic acid	Sigma–Aldrich	≥97%
1293	Tridec-1-ene	Sigma–Aldrich	96%
1300	Tridecane	Sigma–Aldrich	99%
1391	(*Z*)-Tetrade-4-cene	THJ	85%
1400	Tetradecane	Sigma–Aldrich	99%
1494	Pentadec-1-ene	Sigma–Aldrich	98%

[1] Kovat's indices calculated on DB-5MS column.

[2] The compound was identified by comparing it with the mass spectrum and Kovat's index of reference compound in natural material on a DB-5MS capillary column (Agilent, 30 m×0.25 mm i.d., 0.25 µm film thickness) with oven temperature of 40 °C for 2 min increasing at 5 °C/min to 300 °C.

[3] Sigma–Aldrich, St. Louis, MO, USA

[4] THJ The compound was synthesized by Tappey H. Jones

[5] Fisher Scientific, Hampton, NH, USA

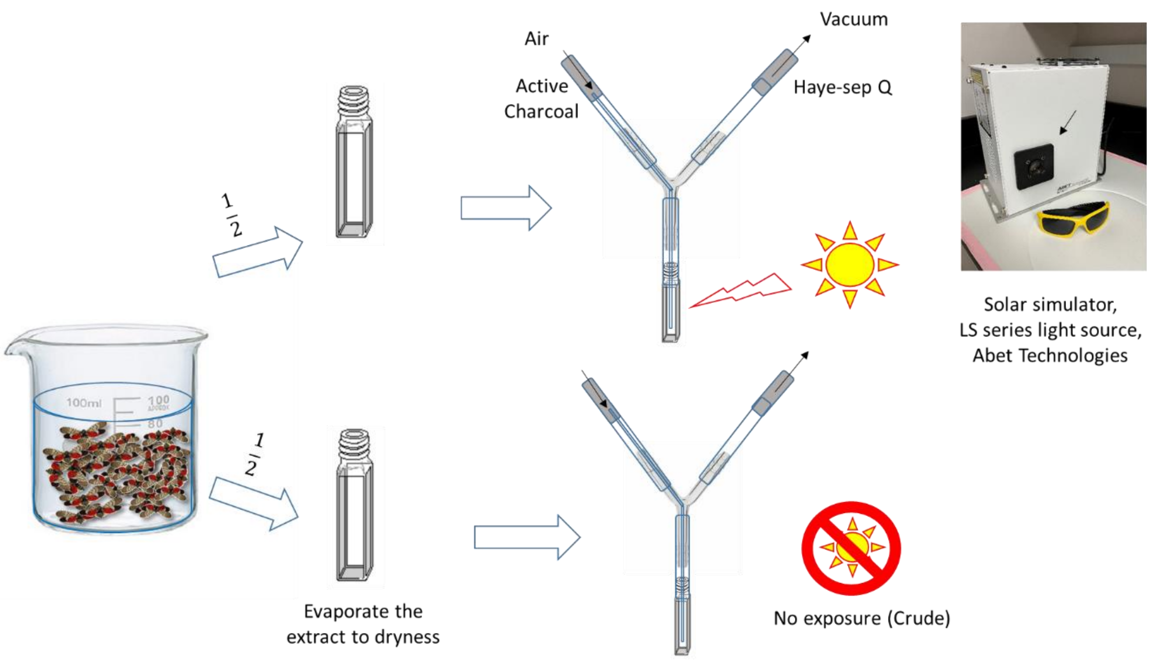

Figure S1. The process of sample preparation for photo-degradation. Whole-body spotted lanternfly extracts were divided into two equal portions, half slated for photo-degradation, and the other half to remain as crude extract (control). Each portion was then evaporated just to the point of dryness in a quartz cuvette. The cuvette vials were either exposed (photo-degraded) or not exposed (crude) to a solar simulator, by placing the cuvette either directly in front of, or away from, the light source (arrow) during which time the headspace body volatiles were collected on HayeSep-Q.

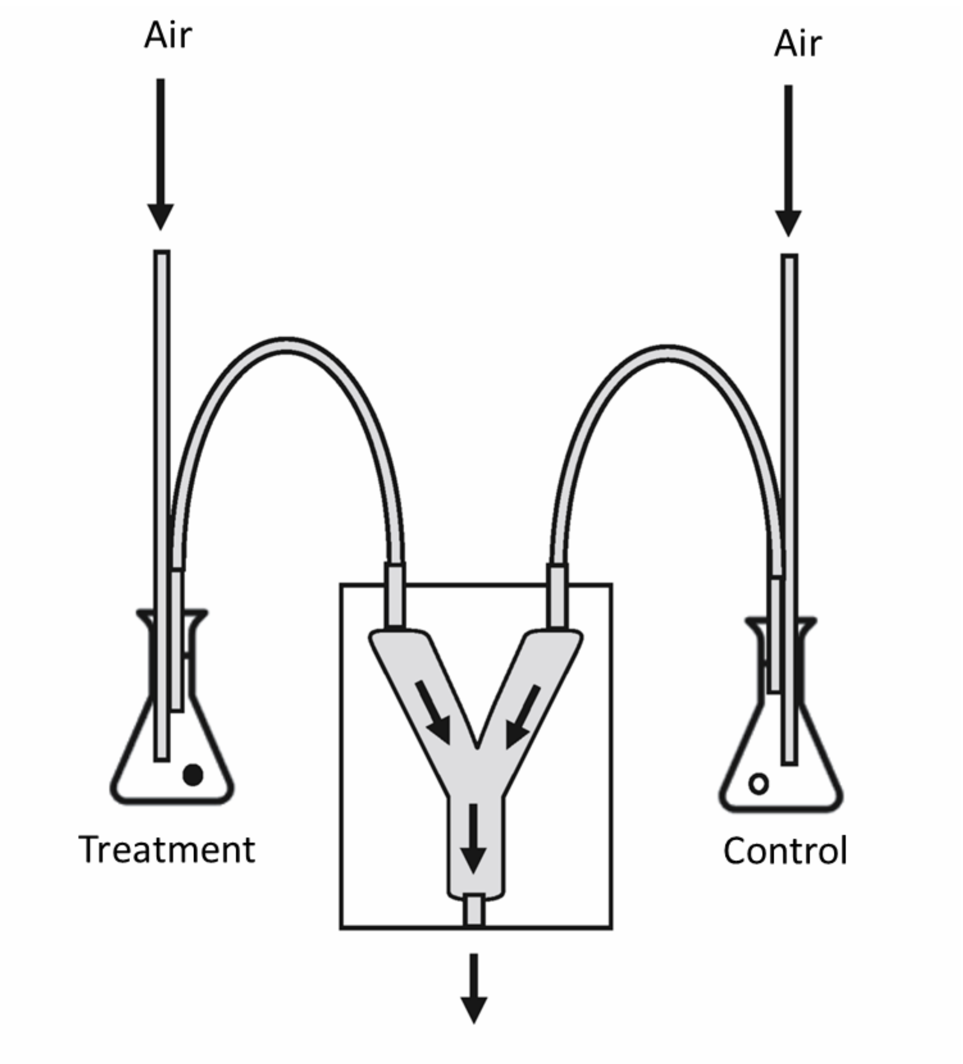

Figure S2. Diagrams of a Y-plate in two-choice bioassays used in the study. Diagram is not to scale. On both sides of the olfactometer, the Charcoal-filtered humidified air passed through a 50-ml glass flask prior to entering the arm of the olfactometer. A lure containing the stimulus or the control was placed in the flasks.

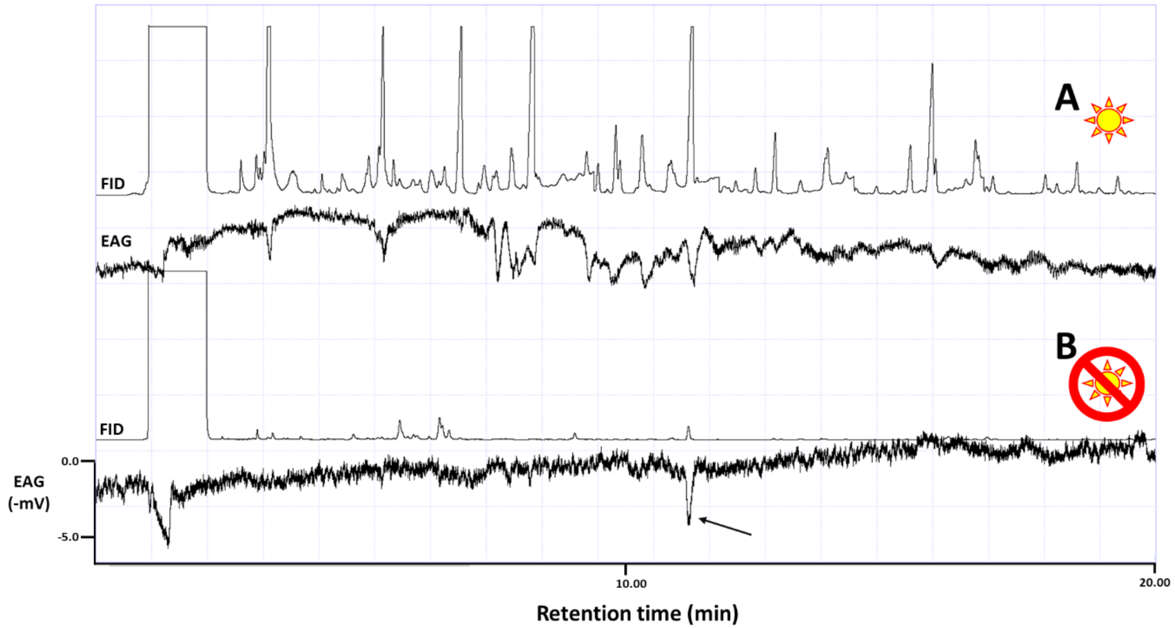

Figure S3. Representative traces from gas chromatography-electroantennographic detection (GC-EAD) analysis of spotted lanternfly whole-body extracts, using male antennae in response to (A) photo-degraded female extract or (B) crude female extract. The top trace shows GC peaks of chemicals eluting to the flame ionization detector (FID), and the corresponding bottom trace (EAG) shows the depolarization of olfactory sensilla in the male antenna. An arrow points to a strong antennal response to nonanal, for example. Photo-degradation process resulted in an increase in both the amount and number of antennal active components compared to those in crude extracts.
